# Mixed Methods Findings from a Stepped Wedge Hybrid Implementation Trial of ATTAIN NAV: A Mental Health Family Navigation Intervention for Autistic Youth

**DOI:** 10.1007/s10803-025-06851-7

**Published:** 2025-05-08

**Authors:** Nicole A. Stadnick, Kassandra Martinez, Felice Navarro, Pollyanna Gomez-Patino, Kimberly Holmquist, Sonya Negriff, Scott Roesch, Isaac Bouchard, Sara Walpole, Romina Espinosa, Sarabeth Broder-Fingert, Miya Barnett, Lauren Brookman-Frazee

**Affiliations:** 1 Department of Psychiatry, University of California San Diego, La Jolla, CA USA; 2 Altman Clinical and Translational Research Institute Dissemination and Implementation Science Center, University of California San Diego, La Jolla, CA USA; 3 Child and Adolescent Services Research Center, San Diego, CA USA; 4 San Diego State University/University of California San Diego Joint Doctoral Program in Clinical Psychology, San Diego, CA USA; 5 Department of Research and Evaluation, Kaiser Permanente Southern California, Pasadena, CA USA; 6 Department of Psychology, San Diego State University, San Diego, CA USA; 7 Department of Psychology, California State Northridge, Northridge, CA USA; 8 Kaiser Permanente Southern California, San Diego, CA USA; 9 Department of Pediatrics, University of Massachusetts Chan Medical School, Worcester, MA USA; 10 Department of Counseling, Clinical, and School Psychology, University of California Santa Barbara, Santa Barbara, CA USA

**Keywords:** Family navigation,autism, Mental health, Stepped wedge, Hybrid implementation effectiveness, Mixed methods

## Abstract

ATTAIN NAV (Access to Tailored Autism Integrated Care through Family Navigation) was delivered by family navigators to promote access to and engagement with mental health services for school-age autistic youth. This study used a mixed method, stepped wedge design to test the effects of family navigation on service and clinical outcomes while gathering information on implementation. Primary care providers from six clinics in California and 56 caregiver-child dyads enrolled in and completed the study. Clinics were randomized to either a technology-enhanced or standard family navigation condition. Caregivers completed assessments at baseline and post about child, family and services outcomes, and a subset participated in a post qualitative interview. Quantitative findings demonstrated improvements in child challenging behavior and parent activation across conditions although these improvements were more pronounced for families in the standard FN condition. At post-intervention, families in the standard FN condition reported higher levels of navigation satisfaction, a shorter time to attend their first mental health appointment, and higher engagement with their navigator. Qualitative findings complemented and expanded the quantitative survey findings. The ATTAIN NAV model of family navigation for autistic children with co-occurring mental health needs demonstrates promising implementation, service, and clinical benefits. **Clinical Trials Registration**. NCT05344378.

## Introduction

Autistic youth commonly experience co-occurring psychiatric conditions requiring access to and engagement with mental health services (Brookman-Frazee et al., 2017; Lai et al., 2019; Levy et al., 2010). Despite the high prevalence of co-occurring mental health needs, youth with autism face barriers to care throughout the help-seeking process (Joshi et al., 2010; Wood et al., 2015). These individuals often face delays in identifying co-occurring mental health needs and following through on recommended referrals (Reaven & Wainer, 2015). Additionally, the mental health workforce is limited by overall volume and specialization in caring for autistic youth (Vohra et al., 2014). The lack of coordinated care between service systems further exacerbates the barriers youth with autism face to engage in needed mental health care (Maddox et al., 2021).

Primary care is a principal point of care for youth that can promote early identification and engagement with mental health services for autistic youth (Jellinek & Murphy, 2021). Integrated healthcare models (i.e., concerted efforts to coordinate care between primary care and specialty care settings) and are promising care models to facilitate mental health care engagement for autistic youth (Asarnow et al., 2015; Farmer et al., 2014). In our prior work, our team co-designed “Access To Tailored Autism Integrated Health Care (ATTAIN)” (Stadnick et al., 2022), which comprised a mental health screening and referral process for autistic youth in pediatric primary care. ATTAIN implementation was evaluated and found to be feasible and acceptable from provider and caregiver perspectives. However, additional caregiver guidance to successfully connect to needed child services and personalized implementation support for healthcare organizations were needed.

Family navigation is an evidence-based care management strategy that has the potential to improve the implementation and effectiveness of integrated healthcare models and successful engagement with needed healthcare services (Feinberg et al., 2021a, 2021b; Godoy et al., 2019). Family navigators can provide a range of valuable resources, ranging from psychoeducation, assistance with navigating barriers to services, and promoting communication between the family and healthcare provider (Godoy et al., 2019). There are several types of family navigation models that vary based on the characteristics of the navigator and the purpose of navigation. For example, clinician-led navigation uses professionals as navigators, whereas peer-led or paraprofessional models rely on individuals who have personal experience with navigating healthcare systems or similar cultural backgrounds (Godoy et al., 2019). Broder-Fingert and colleagues (2020) identified and defined the core components of family navigation for caregivers of youth with autism. Eleven core components were defined and grouped into three domains: Training Supervision, Navigator Activities, and Navigator Tools. Training and Supervision ensures navigators receive initial training, ongoing supervision and case review, and fidelity monitoring. Navigator Activities address direct engagement between navigators and families, including referrals to navigation, linguistic and cultural brokering, completing encounters, barrier identification, emotional support, and care coordination. Navigator Tools assist navigators in supporting families by providing a navigator workbook (e.g. templates for family-specific action plans, training materials, psychoeducational resources) and a navigator checklist.

In response to need for greater caregiver service guidance and organizational implementation support for integrated autism mental health care, our team enhanced ATTAIN by adding a family navigation component, referred to as ATTAIN NAV. Family navigation for autism was adapted for ATTAIN using a series of qualitative co-design interviews with caregivers of autistic youth, primary care and mental health clinicians, and health informatics specialists. The current study reports mixed methods findings from a stepped-wedge implementation trial of ATTAIN NAV evaluating technology enhancements in pediatric primary care settings.

## Methods

### Setting

Kaiser Permanente Southern California (KPSC) is an integrated healthcare delivery system serving over 4.6 million racially and socio-economically diverse members, including 1.5 million children. There are 15 medical centers (including inpatient and outpatient services) and 233 medical offices (outpatient). Health care at KPSC is coordinated through an integrated electronic health record (EHR) system that captures comprehensive information on the care members receive at KPSC-owned and contracting facilities. KPSC also obtains claims data on any out-of-network care that members receive.

### Study Conditions

There were two study conditions: technology-enhanced FN and standard FN. The technology-enhanced condition included three components: a KP.org messaging (Patient-Facing) capabilities, Twilio/REDCap (Patient-Facing) messaging, and an enrollment dashboard (Provider-Facing). The purpose of the KP.org messaging system was to share resources (e.g., websites, handouts) discussed by navigators in family sessions that are not easily sent in space-limited communication. The purpose of the Twilio/REDCap messaging was to send affirmations and reminders to families about upcoming appointments, homework assignments, and use of navigation skills. The enrollment dashboard was designed to update enrolled PCPs about the status of their referred families. Standard FN was delivered without the technology-enhanced components.

### Study Design

**Fig. 1:**
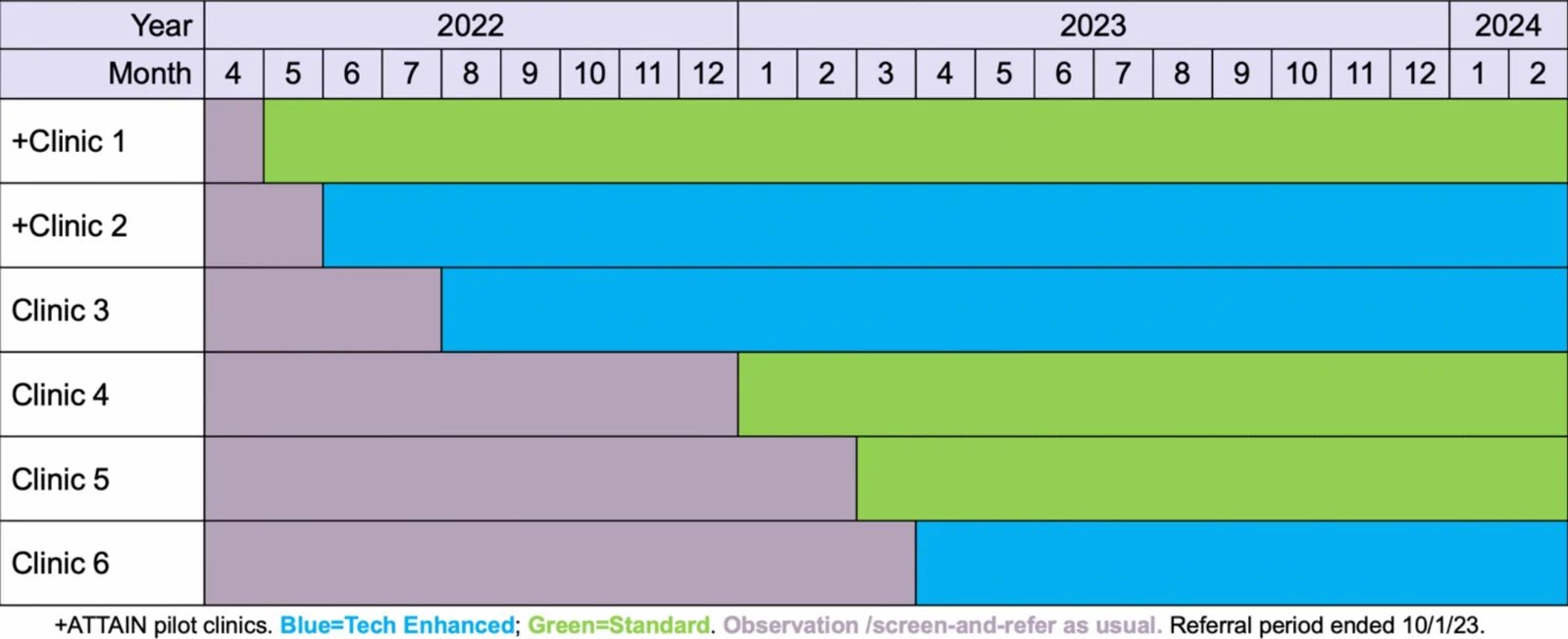
ATTAIN NAV Cluster randomized stepped wedge design

This study used a mixed method, modified cluster randomized stepped wedge hybrid type 1 effectiveness implementation trial design to test the effects of technology-enhanced vs standard FN on service and clinical outcomes while gathering information on FN implementation. (see Fig. 1). A virtual random coin toss was used to determine the condition for the Step 1 Clinics, which participated in the ATTAIN pilot. Heads was the Technology-Enhanced condition and Tails was the standard FN condition. A random order generator (RANDOM.ORG) was then used to determine the step for the remaining four clinics. Clinics were assigned to a FN condition every 3 months until all clinics were in a FN condition. Data collection was continuous at all clinics through the trial so that each cluster contributed data during observation and intervention periods.

### Participants

**Fig. 2:**
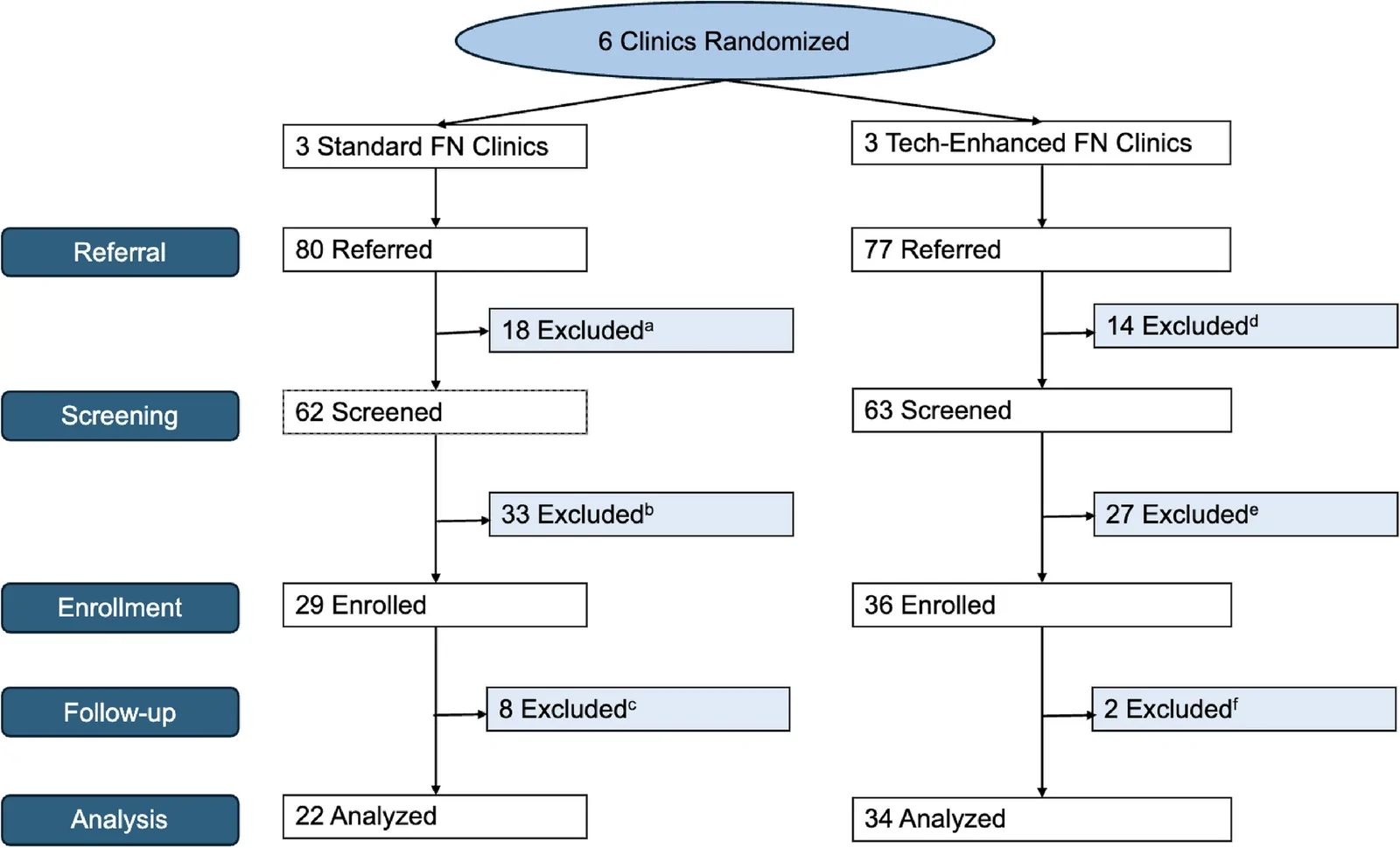
ATTAIN NAV CONSORT flow diagram. **a**. 6 = Unable to reach for screening call; 6 = Able to reach for screening call, but declined screening​; 5 = Not eligible due to age; 1 = Not eligible due to insurance status. **b**. 27 = PSC-17 did not meet cutoff​; 6 = PSC-17 met cutoff, but declined participation​. **c**. 8 = Discontinued participation. **d**. 5 = Unable to reach for screening call; 8 = Able to reach for screening call but declined screening​; 1 = Caregiver preferred language not English or Spanish. **e**. 18 = PSC-17 did not meet cutoff​; 9 = PSC-17 met cutoff, but declined participation. **f**. Discontinued participation

A total of 21 primary care providers from six clinics and 65 caregiver-child dyads who were receiving care from a participating primary care provider enrolled in the study. A total of 157 families were referred to the study. Following screening, 57 were not eligible and another 35 declined participation. Of those families enrolled, 34 were referred by their child’s PCP and 31 were self-referred. A total of 56 caregiver-child dyads were included in analyses. Figure 2 shows the CONSORT flow diagram.

### Procedures

A total of three family navigators were hired and trained in ATTAIN NAV by the university partner (University of California San Diego). Training included an 8-h initial training and ongoing bi-weekly supervision meetings with the PI who is a licensed psychologist, a clinical psychology doctoral candidate, and a licensed marriage and family therapist at KPSC.

The navigators were hired through a university job search with the following hiring criteria: professional experience in health education, service navigation or related field, bilingual in Spanish and English, and preference for caregivers of neurodiverse children. The positions were part-time (25%). There were two original female navigators hired. One original navigator had worked for the County of San Diego as a social worker, was in the process of finishing her Master of Public Health degree and was a mother of a child with developmental needs. The second original navigator had worked as a Spanish-speaking medical interpreter for several years. She left the study after six months to pursue a full-time position. The third navigator who replaced the second original navigator held a Master Degree of Public Health, had worked for as a Health Educator for a local non-profit community clinic, and was a mother of a child with developmental needs. One navigator was assigned to each navigation condition.

All providers from each of the participating clinics were invited to enroll. Eligibility for providers was that they be employed at the participating clinic at least part-time and were expected to retain employment for at least 6 months. Eligible families were identified through a referral by their child’s pediatrician using an Epic smartphrase or self-referral in response to a personalized email invitation that described the study. Within 1–7 business days of a referral, a family navigator contacted the caregiver to screen for eligibility and complete the Pediatric Symptom Checklist-17 (PSC-17) (Murphy et al., 2016). The PSC-17 measures internalizing symptoms (e.g., anxiety), externalizing symptoms (e.g., aggression), and attention problems (e.g., hyperactivity). If the child’s PSC-17 Total Score was 15 or higher, the child was between 4 and 16 years old and had a documented autism diagnosis (based on referring provider and caregiver validation), they were eligible to participate in the ATTAIN NAV study. Family navigators reviewed the informed consent with eligible families and, if the families provided verbal and written consent, navigation commenced for up to four months. The study was designed to accommodate 30 primary care providers and 160 families based on navigator caseloads of 4 to 6 families and power calculations to detect predicted effects.

The initial stage of ATTAIN NAV was developing the Family Development Plan which consisted of 1–3 SMART (Specific, Measurable, Achievable, Relevant, and Time-bound) goals to support their child’s behavioral and mental health needs. These goals were collaboratively set and could include service navigation to and engagement with autism-specific services, school-based programs, socio-recreational programs, and parent mental health resources. Navigators met approximately weekly with each assigned family for 15 to 60 min via phone, Zoom, or in-person in the family’s home or a community setting. Although family navigation was not a 24 h service, the FNs were flexible to accommodate family schedules during the work week (Monday-Friday), including evenings. Examples of FN activities included three-way calls between the caregiver and mental health service representatives to schedule appointments, guiding caregivers through submitting intake paperwork for developmental disability services, in home support services, and family medical leave requests, and role-playing with caregivers how to advocate for ABA and school-based services for their child in upcoming provider meetings.

Upon completion of the SMART goals or after four months, a final session was scheduled with the family. In the final session, the goals of family navigation were summarized along with strengths and barriers that the family overcame during the family navigation process. The family navigator addressed any pending items or questions from the family and the session was finalized by thanking the family for their participation in the study.

### Measures

Quantitative and qualitative measures were used to assess ATTAIN NAV clinical and implementation outcomes. Provider and child/family demographic characteristics were collected at baseline. Caregivers received $20 for completing the baseline survey, $20 for completing the post-survey, and $25 for participating in an individual post-interview. Table 1 displays the quantitative outcome measures used.

**Table 1:** Quantitative measures

Purpose	Construct	Measure	Source	Timeframe	
				Baseline	Post
Outcomes	Implementation	Navigation Satisfaction Tool	Caregiver		X
		Navigator fidelity: ≥ 80% mastery of all family navigator components	Navigator		X
	Service	Time to First Mental Health Appointment	Navigator		X
		Services Referred-To	Navigator		X
	Child Clinical	Child Mental Health symptom improvement (ECBI)	Caregiver	X	X
	Family	Parent Activation Measure for Developmental Disabilities	Caregiver	X	X
Change Mechanisms	Navigator Engagement	Adapted Parent Participation Engagement Measure	Caregiver		X
		Number of Navigator-Family Contacts	Navigator		X
		Average Length of Successful Contacts	Navigator		X

### Implementation Outcomes

#### Navigation Satisfaction Tool

Caregivers’ satisfaction with the relationship with the navigator and with the referred services to which the navigator facilitated access was assessed with the 30-item caregiver-report NAVSAT (Fishman et al., 2018). Items 1–12 assess respondents’ *satisfaction with family navigation* on a 7-point Likert scales ranging from “extremely dissatisfied” to “extremely satisfied”. Respondents also report how often meetings with the navigator were held, rated on a 7-point Likert scale ranging from “less than once every two months” to “more than once per week” and how likely they are to recommend the service to family and friends, rated on a 7-point Likert scale ranging from “very unlikely” to “very likely.” Respondents are first asked to indicate which services they were referred to on a dichotomous yes/no scale (e.g., community mental health, ABA, physical therapy). Items 13–23 do not contribute to the overall score though they help ascertain which services were engaged with. Items 24–30 assess respondents’ *satisfaction with recommended services*. Respondents indicate their satisfaction or perceived effectiveness of the services on a 7-point scale ranging from “extremely ineffective” to “extremely effective” or “extremely dissatisfied” to “extremely satisfied”. Caregivers completed the NAVSAT at post. Mean scores for each subscale were computed. Internal consistency for the subscales was excellent (α = 0.98 for navigation-level satisfaction, α = 0.97 for service-level navigation).

### Fidelity

Navigator fidelity was assessed using fidelity procedures (observational and self-report) from the navigator training curriculum used in previous autism-specific family navigation studies (Broder-Fingert et al., 2019, 2020). Navigators provided self-report fidelity ratings at three time-points: the Family Plan Development session (first navigation session), a follow-up session at the midpoint of family navigation, and the final session. Fidelity was defined as demonstrating mastery on at least 80% of components (e.g., navigator introduces purpose of navigation during initial contact, conducts assessment of barriers to attendance of first mental health appointment). A mean fidelity rating across the three sessions was computed for each family.

### Service Outcomes

#### Family Navigation Contact Logs

Contact logs were completed by navigators after every session. Contact time/date, mode of communication, length of contact (in minutes), reason for contact, identified barriers to following through on action items (if applicable), and other clinically relevant notes were tracked in the contact log.

### Time to First Mental Health Appointment

The difference in days between the Family Plan Development Session date and date of the child’s first appointment with the Kaiser Permanente Southern California Psychiatry department (if applicable) were calculated. These data were abstracted from the family navigation contact logs.

### Number of Referred-To Services

Navigators tallied how many services each participant family was referred to and engaged with during their time receiving ATTAIN NAV. Examples of services families were referred to (in addition to Psychiatry) included community mental health services, school/special education services, Applied Behavior Analysis, and caregiver advocacy services. These data were abstracted from the family navigation contact logs.

### Clinical Outcomes

#### Eyberg Child Behavior Inventory

Changes in child mental health symptoms were assessed with the ECBI (Eyberg & Pincus, 1999), a 36-item caregiver-report measure that assesses the frequency and intensity of child behavior problems. The ECBI evaluates disruptive behavior among children ages 2–16 years old, measuring oppositional behaviors, aggressive conduct, and attention difficulties. Each item is rated in two ways: (1) the frequency of the behavior, rated on a 7-point scale from “never” to “always” and (2) whether the behavior is perceived as a problem by the respondent, rated on a dichotomous “yes”/”no” scale. The ECBI results in two subscales – Intensity and Problem – that are converted to t-scores. T-scores that are ≥ 60 are the cutoff for clinical significance. The ECBI was completed by caregivers at baseline and post.

### Parent Activation Measure for Developmental Disabilities

Changes in caregiver knowledge, skill, and confidence to manage their child were assessed with the 13-item caregiver-report PAM-DD (Ruble et al., 2018). Respondents rate each item on a 4-point Likert scale ranging from “strongly disagree” to “strongly agree.” For the current study, scores were derived using guidelines delineated by Yu et al. (2022) who examined the psychometrics of the PAM-DD in autism. The first subscale includes items related to the motivation and ability to actively intervene for one’s child (e.g., ability to reduce problems, handle services, implement home treatments, understand behavior causes, use available treatments, implement recommendations, prevent problems, figure out solutions, and maintain changes). The second subscale contains items related to parental knowledge, cooperation, and agreement with treatment (e.g., responsibility for child’s behavior, participation with treatment role, knowledge about reasons for medication used, the ability to communicate concerns). The PAM-DD was completed by caregivers at baseline and post. Internal consistency for the subscales was acceptable (Subscale 1 baseline α = 0.67, Subscale 2 baseline α = 0.88; Subscale 1 post α = 0.77, Subscale 2 post α = 0.95).

### Change Mechanisms

#### Adapted Parent Participatory Engagement Measure

Parent engagement was assessed using the 5-item, caregiver-report, PPEM (Haine-Schlagel et al., 2016). Each item assesses the frequency that a parent engaged in a participation behavior (e.g., asked questions, provided input, agreed with the plan) during a mental health appointment. Items are rated on a 5-point scale from “not at all” to “very much”. The items were minimally adapted to assess caregiver engagement during interactions with the navigator. Caregivers completed the PPEM at post. A mean score was used in analyses. Internal consistency was excellent (α = 0.91).

### Number of Navigation-Family Contacts

The number of contact attempts (e.g., navigation sessions, texts, voicemail messages) between the navigator and each family during the interval between PCP referral to ATTAIN NAV and the conclusion of ATTAIN NAV was calculated. These data were abstracted from the family navigation contact logs.

### Average Length of Successful Contacts

The average length of successful contacts was calculated for each family. A successful contact was when the family and Navigator interacted for at least one minute by phone, Zoom, or in-person. These data were abstracted from the family navigation contact logs.

### Qualitative Interviews

At the conclusion of the trial, enrolled families who had completed ATTAIN NAV were invited to participate in an individual semi-structured interview to share their experiences with ATTAIN NAV. Interview guides were constructed to support a semi structured funnel approach where each section started with a broad question and progressively narrowed focus on specific aspects of navigation.

The caregiver interview guide began with a brief discussion of caregivers’ experiences receiving family navigation services for their child and concluded with inquiry about potential changes to specific aspects of navigation. Please see Appendix A for the interview guide.

### Data Analyses

#### Quantitative

First, descriptive statistics were performed to characterize the sample and identify unusual data patterns. Multilevel linear modeling was used as the primary statistical technique to account for the nested data structure: repeated measures [level-1] nested within PCP/navigator/child [level-2] nested within primary care clinic [level-3]). All analyses used an intent-to-treat approach. Covariates including baseline scores were included to account for nonequivalence at baseline and to reduce bias. All analyses of caregiver/child and navigator level were conducted using generalized estimating equation models adjusting for clinic. Moreover, the time trend for the steps was accounted for by creating dummy-coded variables with the 1 st step serving as the reference time-point; these variables were evaluated as fixed effects in the target models. Finally, there were significant differences in the number of caregivers who were Hispanic as a function of clinic. This variable was entered in all models as a covariate (fixed effect).

### Qualitative

All interview data were transcribed from audio recordings. The final codebook included 17 codes that were defined and applied to the interview data. A team of three research members (the PI, a family navigator, and a graduate research assistant) first independently applied the codebook to each transcript. Subsequently, the coding team met to review their independent codes and reach consensus on the final codes for each transcript. Directed content analysis applying constant comparison methodology (Assarroudi et al., 2018; Hsieh et al., 2005) was used to identify qualitative themes across the interviews.

### Mixed-Methods

Following interview data analysis, the research team connected the quantitative and qualitative datasets for the purposes of complementarity and expansion (Palinkas et al., 2011).

### Community Involvement

The family navigation intervention, ATTAIN NAV, was co-designed and refined with caregivers of autistic children, healthcare providers, and healthcare system staff.

## Results

### Baseline Demographic Data

Of the 21 enrolled PCPs, 71.4% (n = 15) identified as female and 23.8% (n = 5) identified as male. In terms of ethnicity, 85.7% (n = 18) identified as non-Hispanic. Regarding professional characteristics, 71.4% had worked in their current healthcare organization for more than 5 years. All providers (n = 21) reported that autistic children represented 10–25% of their patient load. The majority (95.2%) reported caring for more than 20 patients a day. A total of 42.9% (n = 9) reported participating in the ATTAIN study, which preceded the current study. Please see Table 2 for more details.

**Table 2:** Primary care provider demographic and professional characteristics (n = 21)

	n	%
Gender		
Male	5	23.8
Female	15	71.4
Chose not to respond	1	4.8
Ethnicity		
Non-Hispanic	18	85.7
Hispanic	2	9.5
Not reported	1	4.8
Years at Organization		
< 1 year	1	4.8
3–10 years	11	47.6
> 10 years	8	38.1
Not reported	2	9.5
Patient Load		
More than 20 a day	20	95.2
16–20 patients a day	1	4.8
Autistic patient caseload		
10–25%	21	100
Participated in prior ATTAIN Study		
Participated	9	42.9
Did Not Participate	12	57.1

Among child participants (n = 56), 82.1% (n = 46) were male and 17.9% (n = 10) were female. In terms of race, 27% (n = 15) reported as White/Caucasian, 20% (n = 11) as Multiracial, 14% (n = 8) as Asian American/Native Hawaiian/Pacific Islander, 5% (n = 3) as Middle Eastern/Northern African, and 4% (n = 2) as Black/African American. In terms of ethnicity, 48% (n = 27) reported as Hispanic. Children were an average of 7.6 years old (*SD* = 2.8).

Among caregiver participants (n = 56), 88% (*n* = 49) identified as female and 13% (*n* = 7) identified as male. In terms of race, 41% (*n* = 23) identified as White/Caucasian, 20% (*n* = 11) as Asian American/Native Hawaiian/Pacific Islander, 7% (*n* = 4) as Multiracial, 7% (*n* = 4) as Middle Eastern/Northern African, and 4% (*n* = 2) as Black. In terms of ethnicity, 43% (*n* = 24) identified as Hispanic. Of those who responded, the most common income category reported was “$60,001 – $105, 000” (*n* = 16, 28.6%). Most caregivers (*n* = 38, 67.9%) reported their highest education level as a bachelor’s degree or higher. Most parents’ reported English as their preferred language (*n* = 54, 96.4%). Please see Table 3 for more details.

**Table 3:** Child and caregiver baseline characteristics (*n* = 56)

	Child		Caregiver	
	*n*	%	*n*	%
Gender				
Female	10	17.9	49	88
Male	46	82.1	7	13
Race				
White/Caucasian	15	27	23	41
Multiracial	11	20	4	7
Asian American/ Native Hawaiian/Pacific Islander	8	14	11	20
Middle Eastern/Northern African	3	5	4	7
Black	2	4	2	4
Not reported	13	23	12	22
Ethnicity				
Non-Hispanic	28	50	32	57
Hispanic	27	48	24	43
Chose not to respond	1	2	0	0
Income				
$0 – $60,000	–	–	4	7.1
$60,001 – $105, 000	–	–	16	28.6
$105,001 – $150,000	–	–	11	19.6
$150,000 +	–	–	10	17.9
Chose not to respond	–	–	15	26.8
Highest Education				
HS/GED	–	–	2	3.6
Associates/Some College	–	–	16	28.6
Bachelor’s Degree	–	–	21	37.5
Master’s Degree	–	–	15	26.8
Doctoral/Medical Degree	–	–	2	3.6
Preferred Language				
English	–	–	54	96.4
Spanish	–	–	2	3.6
PSC-17 Total Score *≧ 15 = elevated	*M* = 19.8; *Range* = 15–29	*SD* = 3.2		

### Implementation Outcomes

#### Navigation Satisfaction

Parent-reported satisfaction with family navigation and with services referred to significantly differed between conditions, with parents in the standard FN condition (S) reporting higher satisfaction with family navigation and referred-to services (*B* = − 0.38 & *B* = − 0.95, respectively) compared to families in the technology-enhanced condition (TE). Families who were patients in clinics randomized to step 1 reported significantly higher satisfaction with family navigation and with services referred to compared to families who were patients at clinics in step 2 (*B* = − 0.72) and step 3 (*B* = − 0.40).

### Navigator Fidelity

On average, navigators achieved fidelity across all rated sessions. Navigator fidelity significantly differed between conditions, with higher navigator fidelity in the standard FN condition compared to the technology-enhanced condition *B* = − 2.96 (S > TE). There were no significant differences in navigator fidelity between RCT steps.

### Service Outcomes

#### Time to First Mental Health Appointment

Thirty-five (63%) enrolled families were referred to KPSC Psychiatry services. Of those, 24 (38%) families attended an appointment with the KPSC Psychiatry department. These families attended their first Psychiatry appointment within an average of 42.6 days (*SD* = 32.5). The number of days between the start of family navigation and families’ first appointment with the KPSC Psychiatry department significantly differed between conditions, with families in the standard FN condition attending their first appointment sooner compared to those in the technology-enhanced condition (*B* = 16.18). Families who were patients at clinics randomized to step 1 took longer to attend their first Psychiatry appointment compared to families whose clinics were randomized to step 2 (*B* = − 33.28) and step 3 (*B* = − 6.88).

### Number of Services Referred to

On average, families were referred to 5.21 (*SD* = 1.9) services. The most referred to services were recreational/social services (*n* = 38), KPSC Psychiatry services (*n* = 35), external Psychiatry services (*n* = 34) and San Diego Regional Center (primary funder of developmental disabilities services in the state) (*n* = 31). The number of services referred to differed by condition, with families in the technology-enhanced condition referred to significantly more services (*B* = 0.96). Families who were patients at clinics randomized to step 1 were referred to significantly more services compared to families who were patients at clinics randomized to step 2 (*B* = − 1.22). There were no differences in the number of services referred to between steps 1 and 3.

### Child Clinical Outcomes

#### ECBI Intensity Scale

The frequency of problem behaviors reported by caregivers significantly decreased for both FN conditions from baseline to post, but the decrease was sharper for families in the standard FN condition (*B* = 3.62) and the t-scores for these children fell below the cutoff for clinical significance at post (*M* = 57.20; *SD* = 8.01). Families who were patients at clinics randomized to step 2 (*B* = − 2.69) and step 3 (*B* = − 4.31) reported greater declines from baseline to post in the frequency of problem behaviors compared to families at clinics randomized to step 1 and the t-scores for these children fell below the cutoff for clinical significance at post (step 2: *M* = 58.79; *SD* = 7.89; step 3: *M* = 56.88; *SD* = 9.45).

### ECBI Problem Scale

Similarly, the extent to which caregivers found the child’s behaviors to be a problem significantly decreased for both FN conditions from baseline to post, but the decrease was sharper for families in the standard FN condition (*B* = 5.54) and the t-scores for these children fell below the cutoff for clinical significance at post (*M* = 56.05; *SD* = 10.39). Families who were patients at clinics randomized to step 2 reported greater decreases from baseline to post in the severity of problem behaviors compared to families at clinics randomized to step 1 (*B* = − 1.81) and the t-scores for these children fell below the cutoff for clinical significance at post (*M* = 57.82; *SD* = 10.58). There were no differences in the severity of problem behaviors over time between steps 1 and 3.

### Family Outcomes

#### PAM-DD Subscale 1

Parent-rated motivation and ability to actively intervene for their child (PAM-DD Subscale 1) significantly increased across both conditions. Parents who received standard FN rated themselves higher in parent ability and motivation to intervene for their child compared to those in the technology-enhanced FN condition (B = − 0.31). Families who were patients at clinics randomized to step 1 rated themselves higher compared to families who were patients at clinics randomized to step 3 (*B* = 0.30). There were no differences between steps 1 and 3.

#### PAM-DD Subscale 2

Parent-rated parental knowledge, cooperation, and agreement with treatment (PAM-DD Subscale 2) significantly increased across both conditions. Parents who received standard FN rated themselves higher in knowledge, cooperation, and agreement with treatment compared to those in the technology-enhanced FN condition (B = − 0.26). Families who were patients at clinics randomized to step 1 rated themselves higher compared to families who were patients at clinics randomized to step 2 (*B* = − 0.08). There were no differences between steps 1 and 3.

### Change Mechanisms

#### PPEM

On average, parent-rated engagement with the navigator was high at post (*M* = 4.35, *SD* = 0.8). There were significant differences between conditions, with families in the standard FN condition reporting higher engagement (*B* = − 0.17). Parent-rated engagement significantly differed between families who were patients in clinics in step 1 compared to step 2 (*B* = − 0.54) and step 3 (*B* = − 0.43).

### Number and Length of Contacts

On average, navigators made an average of 26.61 (*SD* = 12.5) contact attempts with enrolled families. When contact attempts were successful (i.e., the parent and navigator interacted for at least one minute), parents and navigators engaged for 22.1 min (*SD* = 8.8). There was a significant difference between conditions, with the navigator in the technology-enhanced condition attempting more contacts and engaging in successful attempts for longer periods of time (*B* = 10.69 and *B* = 10.79, respectively). The number of contact attempts differed for families who were patients in clinics in step 1 compared to step 2 (*B* = − 3.35) and step 3 (*B* = − 9.51). Please see Table 4 for full multi-level modeling results.

### Qualitative Findings

The qualitative findings from caregiver interviews (n = 14) coalesced around three primary thematic domains. The three primary thematic domains are described below along with illustrative quotes.

### Theme #1: Caregiver Benefits from FN.

Caregivers shared multiple benefits from receiving FN through ATTAIN NAV. One primary benefit was the emotional and peer support provided by navigators. Caregivers often expressed that the navigator-caregiver relationship fostered a sense of accountability for the caregiver to seek out services or complete tasks aligned with family goals. One caregiver shared,

*“So it was really nice to have somebody calling me once a week and like asking me if there's anything like, I needed help with or just kind of like helping me navigate this disability and care for my child”. – Caregiver A, Standard Family Navigation*

A second benefit that caregivers shared was gaining advocacy skills and feeling empowered to best help their child access different services. One caregiver shared,

*“And with (Family Navigator), she actually, like, modeled for me or showed me how to access the resources.” –Caregiver B, Technology-Enhanced Family Navigation*

A third benefit was the increased knowledge of specific autism, mental health, health, advocacy, and caregiver support (e.g., respite, in home support services) resources needed for their child. As noted by one caregiver,

*“And so, it was a really perfect timing for me because I was having a challenge without ABA therapy. And so, she helped me navigate that and she just kind of like, all the information about my kid and like what we're managing”. – Caregiver B, Technology-*Enhanced Family Navigation

A final benefit of FN was reduced caregiver stress attributed, in part, because their navigator acted as a reliable advocate. This allowed caregivers to spend more time supporting their child in other areas. One caregiver explained,

*“Yeah, I would say [navigation] definitely lowered my stress, because they helped me navigate through the resources that I did want to use and helped me research...find more information. And I guess in return, that does lower my stress, because I have less things for me to do, as far as like research and stuff”. – Caregiver C, Standard Family Navigation*

### Theme #2: Caregiver Experiences with FN.

Caregivers appreciated the flexibility of ATTAIN NAV with respect to appointment times and meeting locations. A caregiver shared,

*“[Navigator] was really flexible. So that wasn't too difficult…she offered to meet in person, but we communicated mostly by phone, via email. So that was pretty accessible to schedule times to talk”. – Caregiver B, Technology-Enhanced Family Navigation*

Caregivers shared differing perspectives on the value of mental health service linkage. While most were grateful to be more aware of their child’s co-occurring mental health needs, some families prioritized linkage to other services. Most caregivers did not observe a change in communication with their pediatrician, but a few caregivers reported learning new tools to better communicate with different service providers to facilitate access to the services they needed for their child. One caregiver shared,

*“So it's hard for me to like, sometimes express myself, so she (Family Navigator) helped me know what to ask the doctors and... how to start a conversation.” – Caregiver D, Technology-Enhanced Family Navigation*

#### Theme #3: Caregiver Recommendations to Refine FN.

Caregivers shared recommendations to refine FN related to the dose of FN and additional service system coordination improvements. Caregivers recommended modifying the frequency and duration of navigation to extend beyond four months with less frequent meetings (e.g., from weekly to monthly). One caregiver explained,

*“Yeah, so that would be interesting if you followed up in a few years and checked in like, “Okay, how's the thing going regarding services and supporting her mental health?” – Caregiver B, Technology-Enhanced Family Navigation*

Some caregivers expressed frustration with the lack of ease with coordinating services across the medical, mental health, and allied healthcare service systems. Caregivers endorsed a need to streamline communication across medical, mental health, and allied service systems. One caregiver described,

*“…So we got ABA, but speech therapy and occupational therapy... speech therapy is still online, you don't have it in person. And then we don't have occupational therapy, even though it was like, requested and it was authorized...we're still missing some therapies” – Caregiver E, Technology-Enhanced Family Navigation*

### Mixed Methods Integration

The qualitative caregiver findings complement and expand the quantitative survey findings in several ways. First, there was complementarity in the navigation satisfaction and engagement findings demonstrated by high levels of navigation-level and service-level satisfaction on the NAVSAT (*Ms* ranged between 5.19–6.52 out of 7 across FN conditions) and the PPEM (*M* = 4.35 out of 5), which were concordant with the global satisfaction from caregiver interviews. The qualitative data expanded the quantitative satisfaction findings by identifying specific areas that caregivers reported navigation benefits including emotional and peer support, reduced caregiver stress, and increased service knowledge and advocacy skills. Second, there is complementarity and expansion regarding the referred services findings. Families were referred to an average of 5.21 services spanning mental health, social/recreational, and developmental disabilities services. This is consistent with the caregiver interviews that described increased knowledge of available services. The caregiver interviews expanded the quantitative findings about referred services through their remarks about the variable interest in pursuing mental health services because of need to prioritize other child and caregiver health and wellbeing services. Third, the qualitative findings uniquely identified areas for future FN refinement to optimize the navigation experience. Specifically, caregivers recommended modifications to the duration and frequency of FN sessions and focused attention on easing coordination and engagement across medical, psychiatric, and allied health service systems. Finally, there were no qualitative findings that explicitly discussed experiences with the technology enhancements, for those in the TE condition.

## Discussion

This mixed method, hybrid implementation-effectiveness stepped wedge study demonstrated the implementation, service, and clinical impacts of two versions of ATTAIN NAV, a family navigation intervention for autistic children with co-occurring mental health needs. The study tested the effects of ATTAIN NAV delivered to 56 families across six randomized primary care clinics through either a technology-enhanced or standard FN condition. Qualitative caregiver interview findings complemented and expanded on quantitative findings.

Quantitative findings demonstrated improvements in child challenging behavior frequency and intensity and parent activation across FN conditions although these improvements were more significant for families in the standard FN condition. At post-intervention, families in the standard FN condition reported higher levels of navigation satisfaction, a shorter time to attend their first mental health appointment, and higher engagement with their navigator. Navigator fidelity in the standard FN condition was also higher but fidelity was met in both conditions, defined as demonstrating mastery on at least 80% of components from observational and self-reported ratings. For families in the technology-enhanced condition, they received more and longer contacts with their navigator and they were referred to more services compared to families in the standard FN condition.

It is noteworthy that the outcomes in the standard FN condition were largely more pronounced compared to the technology-enhanced FN condition. Although families in the technology-enhanced condition received a higher dose of FN and more referred-to services, our findings suggest that these may not be necessary for beneficial outcomes. Offering additional services to families may overwhelm them and shifts caregivers’ attention away from mental health services. This phenomenon has been documented in other parenting intervention studies (Chaffin et al., 2004). Although it was beyond the scope of the study to investigate cost of FN implementation, these findings suggest that there may be cost savings in a standard FN model. The technology enhancements directed towards caregivers in that condition were to enhance communication modalities between the FN, family, and other healthcare providers yet these do not seem to be critical for successful and satisfactory service navigation.

With respect to differences in RCT step, families whose children were patients in step 1, which were the two clinics that also participated in the ATTAIN trial (predecessor to ATTAIN NAV), reported higher navigation satisfaction, longer times to attend their first Psychiatry appointment, more referred services, higher parent activation, higher parent-navigator engagement, and a greater number of FN contacts. Since step 1 clinics (clinics 1 & 2) transitioned earlier to the standard or technology-enhanced conditions, caregivers may have been more familiar with their navigator and navigation services, possibly explaining greater navigator satisfaction, more referred services, and increased parent activation, parent-navigator engagement, and FN contacts. Families whose children were patients in step 2 reported significantly greater changes in child challenging behaviors from baseline to post compared to families whose children with patients in step 1 and step 3 clinics. It is possible that improvements in challenging behaviors for step 2 clinics were more pronounced because of a higher proportion of participants with severe symptoms.

Qualitative findings from caregiver interviews confirmed quantitative findings regarding global satisfaction with FN, increased knowledge and confidence to advocate for their child’s service needs, and the diversity of service needs their child and family required. The qualitative findings uniquely expanded on the quantitative findings through the specific descriptions of FN benefits such as reduced caregiver stress and emotional and peer support, and the recommended areas for targeted refinement of FN. In addition, the qualitative findings may help explain the differences in the effects of FN condition. For example, caregivers who participated in a post qualitative interview suggested that fewer contacts but over a longer period to address developmental changes and service needs of their child may be an important FN refinement moving forward. Although the technology-enhanced FN delivered a higher frequency of FN contacts, families may be better suited to meaningfully engage in and benefit from FN at a lower but more prolonged dose. Interestingly, caregiver interviews did not explicitly discuss experiences, positive or negative, with the technology enhancements for those in that condition.

The ATTAIN NAV findings are consistent with the small but growing body of autism FN research (DiGuiseppi et al., 2021; Feinberg et al., 2021a, 2021b; Mazurek et al., 2024; Roth et al., 2016). Although the existing autism FN research has focused on closing the gaps between referral and service access for initial autism diagnostic evaluation and early intervention, there are overarching commonalities in findings. For example, in their evaluation of embedded primary care provider training with FN, Mazurek and colleagues (2024) reported that most families required ongoing support through FN and needed support to follow-up with a range of service referrals. Aligned with the literature (Crossman et al., 2020) families have also reported reduced stress from participating in autism FN (Table 4).

**Table 4:** Multi-level modeling findings

Outcome	Construct	Descriptive Statistics											Inferential Statistics				
		Condition				RCT Step							Condition	RCT Step			
Implementation	Navigation Satisfaction Tool-Navigation	TE: *M*=6.17 *SD*=0.93		S: *M*=6.52 *SD*=0.76		1: *M*=6.55 *SD*=0.54			2: *M*=6.00 *SD*=1.05			3: *M*=6.34 *SD*=0.96	*B* = − .38 (S>TE)	*B* = − .72 (1>2)			*B* = − .40 (1>3)
	Navigation Satisfaction Tool-Service	TE: *M*=5.19 *SD*=1.31		S: *M*=6.20 *SD*=1.13		1: *M*=5.34 *SD*=1.60			2: *M*=5.55 *SD*=1.18			3: *M*=5.97 *SD*=1.09	*B* = − .95 (S>TE)	NS			
	Navigator Fidelity	TE: *M*=93.39 *SD*=6.59		S: *M*=95.57 *SD*=5.31		1: *M*=93.99 *SD*=5.82			2: *M*=94.14 *SD*=5.84			3: *M*=94.92 *SD*=6.93	*B* = − 2.96 (S>TE)	NS			
Service	Time to first MH Appointment	TE: *M*=46.44 *SD*=37.66		S: *M*=38.00 *SD*=18.15		1: *M*=51.50 *SD*=39.43			2: *M*=23.60 *SD*=8.56			3: *M*=43.33 *SD*=30.39	*B* = 16.18 (TE>S)	*B* = − 33.28 (1>2)	*B* = − 6.88 (1>3)		
	Services Referred-To	TE: *M*=5.08 *SD*=2.35		S: *M*=4.03 *SD*=2.31		1: *M*=4.67 *SD*=2.43			2: *M*=4.00 *SD*=2.22			3: *M*=5.14 *SD*=2.41	*B* = 0.96 (TE>S)	*B* = − 1.22 (1>2)			
Child Clinical	ECBI Problem Scale	BL		Post		BL			Post								
		TE: *M*= 63.45 *SD*=10.17	S: *M* = 64.95 *SD* = 5.64	TE: *M* = 61.16 *SD* = 10.20	S: *M* = 56.05 *SD* = 10.39	1: *M* = 64.16 *SD* = 9.22	2: *M* = 64.00 *SD* = 8.39	3: *M* = 63.94 *SD* = 8.84	1: *M* = 60.26 *SD* = 10.51	2: *M* = 57.82 *SD* = 10.58	3: *M* = 58.88 *SD* = 10.88		*B* = 5.54 (TE>S)	*B* = − 1.81 (1>2)			
	ECBI Intensity Scale	BL		Post		BL			Post				*B* = 3.62 (TE>S)	*B =− 2.69 *(1>2)		*B* = − 4.31 (1>3)	
		TE: *M* = 61.18 *SD* = 9.09	S: *M* = 63.68 *SD* = 6.24	TE: *M* = 60.75 *SD* = 9.75	S: *M* = 57.20 *SD* = 8.01	1: *M* = 62.79 *SD* = 9.14	2: *M *= 61.78 *SD* = 7.82	3: *M *= 61.89 *SD* = 7.74	1: *M* = 61.78 *SD* = 9.63	2: *M* = 58.79 *SD* = 7.89	3: *M* = 56.88 *SD* = 9.45						
Family	PAM-DD Subscale 1	BL		Post		BL			Post				*B* = − .31 (S>TE)	*B* = 0.30 (1>3)			
		TE: *M* = 3.45 *SD* = 0.48	S: *M* = 3.49 *SD* = 0.65	TE: *M* = 3.59 *SD* = 0.44	S: *M* = 3.83 *SD* = 0.29	1: *M* = 3.43 *SD* = 0.56	2: *M* = 3.34 *SD* = 0.69	3: *M* = 3.61 *SD* = 0.42	1: *M* = 3.72 *SD* = 0.34	2: *M* = 3.84 *SD* = 0.26	3: M = 3.51 *SD* = 0.53						
	PAM-DD Subscale 2	BL		Post		BL			Post				*B* = − .26 (S>TE)	*B* = − 0.08 (1>2)			
		TE: *M* = 3.24 *SD* = 0.49	S: *M* = 3.19 *SD* = 0.67	TE: *M* = 3.43 *SD* = 0.57	S: *M* = 3.66 *SD* = 0.46	1: *M* = 3.24 *SD* = 0.54	2: *M* = 3.03 *SD* = 0.70	3: *M* = 3.36 *SD* = 0.42	1: *M* = 3.49 *SD*= 0.63	2: *M* = 3.64 *SD* = 0.38	3: 3.44*M* = *SD* = 0.56						
Change Mechanisms	PPEM	TE: *M* = 4.23 *SD* = 0.84		S: *M* = 4.35 *SD* =0.65		1: *M* = 4.52 *SD* = 0.48		2: *M* = 4.07 *SD* = 0.76	3: *M *= 4.19 *SD* = 0.96				*B* = − .17 (S>TE)	*B* = − .54 (1>2)		*B* = − .43 (1>3)	
	Number of Navigator-Family Contacts	TE: *M* = 29.78 *SD* = 14.13		S: *M* = 18.79 *SD* =5.80		1: *M* = 27.63 *SD* = 15.83		2: *M* = 21.70 *SD* = 8 .32	3: *M* = 24.76 *SD* = 10.98				*B* = 10.69 (TE>S)	*B* = − 3.35 (1>2)		*B* = − 9.51 (1>3)	
	Average Length of Successful Contacts	TE: *M* = 25.44 *SD* = 9.06		S: *M* = 14.78 *SD* =5.81		1: *M* = 18.86 *SD* = 6.90		2: *M* = 19.88 *SD* = 8.39	3: *M* = 23.53 *SD* = 12.14				*B* = 10.79 (TE>S)	*NS*			

*TE* Technology-enhanced navigation, *S* Standard navigation, *NS* Not significant, *BL* Baseline. *p* <.05 for reported beta coefficient

This study has notable strengths and limitations. The strengths include the mixed method study design, pragmatic application of the stepped wedge design, and the robust data collection. Limitations include implementation challenges that occurred because this study was conducted during the COVID-19 pandemic, which required a pause in primary care services that disrupted recruitment, navigator turnover that required a reduction in research activities to train the new navigator, and primary reliance on self-reported measures except for navigator fidelity. Navigator differences and turnover may also contribute to differences between conditions. To address some of these challenges, the research team developed a longitudinal adaptation tracking system to document all planned and unplanned modifications to the study design, FN delivery, and research activities. Another limitation is that our participants reported a somewhat homogenous socioeconomic status (based on educational attainment and household income) that may not represent families experiencing more socioeconomic distress. This is likely the case because participating families were largely privately insured by the participating healthcare organization. Socioeconomic factors may have impacted abilities for families to engage with family navigation. This is a clear area for recommended future research to more precisely unpack the needed socioeconomic and systemic resources for optimal family navigation engagement.

To conclude, the ATTAIN NAV model of family navigation for autistic children with co-occurring mental health needs demonstrates promising implementation, service, and clinical benefits. Next steps are to examine primary care provider perspectives, determine additional refinements needed for scaling and sustaining ATTAIN NAV in additional pediatric service settings, and understand the limitations of the technology-enhanced condition.

## References

[CR1] Asarnow, J. R., Rozenman, M., Wiblin, J., & Zeltzer, L. (2015). Integrated medical-behavioral care compared with usual primary care for child and adolescent behavioral health: A meta-analysis. *JAMA Pediatrics,**169*(10), 929–937. 10.1001/jamapediatrics.2015.1141. PMID: 26259143. 26259143

[CR2] Assarroudi, A., Heshmati Nabavi, F., Armat, M. R., Ebadi, A., & Vaismoradi, M. (2018). Directed qualitative content analysis: The description and elaboration of its underpinning methods and data analysis process. *Journal of Research in Nursing,**23*(1), 42–55. 10.1177/1744987117741667. Epub: 2018 Jan 10 PMID: 34394406 PMCID: PMC7932246. 34394406 PMC7932246

[CR4] Broder-Fingert, S., Qin, S., Goupil, J., Rosenberg, J., Augustyn, M., Blum, N., Bennett, A., Weitzman, C., Guevara, J. P., Fenick, A., Silverstein, M., & Feinberg, E. (2019). A mixed-methods process evaluation of family navigation implementation for autism spectrum disorder. *Autism,**23*(5), 1288–1299. 10.1177/1362361318808460. Epub 2018 Nov 8 PMID: 30404548 PMCID: PMC6742480. 30404548 PMC6742480

[CR5] Broder-Fingert, S., Stadnick, N. A., Hickey, E., Goupil, J., Diaz Lindhart, Y., & Feinberg, E. (2020). Defining the core components of family navigation for autism spectrum disorder. *Autism,**24*(2), 526–530. 10.1177/1362361319864079 31311287 PMC6962571

[CR6] Brookman-Frazee, L., Stadnick, N., Chlebowski, C., Baker-Ericzén, M., & Ganger, W. (2017). Characterizing psychiatric comorbidity in children with autism spectrum disorder receiving publicly funded mental health services. *Autism,**22*(8), 938–952. 10.1177/1362361317712650 28914082 PMC6491206

[CR8] Chaffin, M., Silovsky, J. F., Funderburk, B., Valle, L. A., Brestan, E. V., Balachova, T., Jackson, S., Lensgraf, J., Bonner, B. L, 2004 Chaffin, M., Silovsky, J. F., Funderburk, B., Valle, L. A., Brestan, E. V., Balachova, T., Jackson, S., Lensgraf, J., Bonner, B. L. (2004). Parent-child interaction therapy with physically abusive parents: efficacy for reducing future abuse reports. Journal of Consulting and Clinical Psychology. 72(3):500-510. 10.1037/0022-006X.72.3.500. PMID: 15279533. 15279533

[CR10] Crossman, M. K., Lindly, O. J., Chan, J., Eaves, M., Kuhlthau, K. A., Parker, R. A., Coury, D. L., Zand, D. H., Nowinski, L. A., Smith, K., Tomkinson, M., & Murray, D. S. (2020). Families’ experiences with family navigation services in the autism treatment network. *Pediatrics,**145*, S60–S71. 10.1542/peds.2019-1895I 32238532

[CR12] DiGuiseppi, C., Rosenberg, S. A., Tomcho, M. A., Colborn, K., Hightshoe, K., Gutiérrez-Raghunath, S., Cordova, J. M., Dooling-Litfin, J. K., & Rosenberg, C. R. (2021). Family navigation to increase evaluation for autism spectrum disorder in toddlers: Screening and linkage to services for autism pragmatic randomized trial. *Autism,**25*(4), 946–957. 10.1177/1362361320974175. Epub 2020 Nov 27 PMID: 33246390 PMCID: PMC8723795. 33246390 PMC8723795

[CR14] Eyberg, S. M., & Pincus, D. (1999). *ECBI & SESBI-R: Eyberg child behavior inventory and Sutter-Eyberg student behavior inventory-revised: Professional manual*. Psychological Assessment Resources.

[CR15] Farmer, J. E., Clark, M. J., Mayfield, W. A., Cheak-Zamora, N., Marvin, A. R., Law, J. K., & Law, P. A. (2014). The relationship between the medical home and unmet needs for children with autism spectrum disorders. *Maternal and Child Health Journal,**18*(3), 672–680. 10.1007/s10995-013-1292-z. PMID: 23793533. 23793533

[CR16] Feinberg, E., Augustyn, M., Broder-Fingert, S., Bennett, A., Weitzman, C., Kuhn, J., Hickey, E., Chu, A., Levinson, J., Eilenberg, J. S., Silverstein, M., Cabral, H. J., Patts, G., Diaz-Linhart, Y., Fernandez-Pastrana, I., Rosenberg, J., Miller, J. S., Guevara, J. P., Fenick, A. M., & Blum, N. J. (2021). Effect of family navigation on diagnostic ascertainment among children at risk for autism: A randomized clinical trial from DBPNet. *JAMA Pediatrics,**175*(3), 243–250. 10.1001/jamapediatrics.2020.5218 33427861 PMC7802008

[CR17] Feinberg, E., Kuhn, J., Eilenberg, J. S., Levinson, J., Patts, G., Cabral, H., & Broder-Fingert, S. (2021). Improving family navigation for children with autism: A comparison of two pilot randomized controlled trials. *Academic Pediatrics,**21*(2), 265–271. 10.1016/j.acap.2020.04.007. Epub 2020 Apr 14 PMID: 32302757 PMCID: PMC7554108. 32302757 PMC7554108

[CR18] Fishman, K. N., Levitt, A. J., Markoulakis, R., & Weingust, S. (2018). Satisfaction with mental health navigation services: Piloting an evaluation with a new scale. *Community Mental Health Journal,**54*(5), 521–532. 10.1007/s10597-017-0201-0. Epub 2017 Nov 16 PMID: 29147951. 29147951

[CR19] Godoy, L., Hodgkinson, S., Robertson, H. A., Sham, E., Druskin, L., Wambach, C. G., Beers, L. S., & Long, M. (2019). Increasing mental health engagement from primary care: The potential role of family navigation. *Pediatrics*. 10.1542/peds.2018-2418 30877145

[CR20] Haine-Schlagel, R., Roesch, S. C., Trask, E. V., Fawley-King, K., Ganger, W. C., & Aarons, G. A. (2016). The parent participation engagement measure (PPEM): Reliability and validity in child and adolescent community mental health services. *Administration and Policy in Mental Health,**43*(5), 813–823. 10.1007/s10488-015-0698-x. PMID:26520104 PMCID:PMC4852156. 26520104 PMC4852156

[CR21] Hsieh, H. F., & Shannon, S. E. (2005). Three approaches to qualitative content analysis. *Qualitative Health Research,**15*(9), 1277–1288. 10.1177/1049732305276687 16204405

[CR22] Jellinek, M., & Murphy, J. M. (2021). Screening for psychosocial functioning as the eighth vital sign. *JAMA Pediatrics,**175*(1), 13–14. 10.1001/jamapediatrics.2020.2005 32687559

[CR23] Joshi, G., Petty, C., Wozniak, J., Henin, A., Fried, R., Galdo, M., Kotarski, M., Walls, S., & Biederman, J. (2010). The heavy burden of psychiatric comorbidity in youth with autism spectrum disorders: A large comparative study of a psychiatrically referred population. *Journal of Autism and Developmental Disorders,**40*(11), 1361–1370. 10.1007/s10803-010-0996-9 20309621

[CR24] Lai, M. C., Kassee, C., Besney, R., Bonato, S., Hull, L., Mandy, W., Szatmari, P., & Ameis, S. H. (2019). Prevalence of co-occurring mental health diagnoses in the autism population: A systematic review and meta-analysis. *The Lancet Psychiatry,**6*(10), 819–829. 10.1016/s2215-0366(19)30289-5 31447415

[CR25] Levy, S. E., Giarelli, E., Lee, L. C., Schieve, L. A., Kirby, R. S., Cunniff, C., Nicholas, J., Reaven, J., & Rice, C. E. (2010). Autism spectrum disorder and co-occurring developmental, psychiatric, and medical conditions among children in multiple populations of the United States. *Journal of Developmental & Behavioral Pediatrics,**31*(4), 267–275. 10.1097/dbp.0b013e3181d5d03b 20431403

[CR26] Maddox, B. B., Dickson, K. S., Stadnick, N. A., Mandell, D. S., & Brookman-Frazee, L. (2021). Mental health services for autistic individuals across the lifespan: Recent advances and current gaps. *Current Psychiatry Reports,**23*(10), 66. 10.1007/s11920-021-01278-0. PMID:34402984 PMCID:PMC8961310. 34402984 PMC8961310

[CR27] Mazurek, M. O., Nevill, R. E., Orlando, K., Page, K., Howard, M., & Davis, B. E. (2024). Integration of family navigation into ECHO autism for pediatric primary care in underserved communities. *Journal of Autism and Developmental Disorders*. 10.1007/s10803-024-06445-9 38954361 PMC12476311

[CR29] Murphy, J. M., Bergmann, P., Chiang, C., Sturner, R., Howard, B., Abel, M. R., & Jellinek, M. (2016). The PSC-17: Subscale scores, reliability, and factor structure in a new national sample. *Pediatrics,**138*(3), e20160038. 10.1542/peds.2016-0038. Epub 2016 Aug 12 PMID: 27519444 PMCID: PMC5005018. 27519444 PMC5005018

[CR30] Palinkas, L. A., Aarons, G. A., Horwitz, S., Chamberlain, P., Hurlburt, M., & Landsverk, J. (2011). Mixed method designs in implementation research. *Administration and Policy in Mental Health and Mental Health Services Research,**38*, 44–53. 10.1007/s10488-010-0314-z 20967495 PMC3025112

[CR31] Reaven, J. & Wainer, A. L. (2015). Children and adolescents with ASD and co-occurring psychiatric conditions: Current trends in intervention. *International review of research in developmental disabilities*, *49*, 45–90. Research findings into practice: a consolidated framework for advancing implementation science. Implementation Science. 10.1186/1748-5908-4-50

[CR32] Roth, B. M., Kralovic, S., Roizen, N. J., Spannagel, S. C., Minich, N., & Knapp, J. (2016). Impact of autism navigator on access to services. *Journal of Developmental & Behavioral Pediatrics,**37*(3), 188–195. 10.1097/DBP.0000000000000261 26890560

[CR33] Ruble, L., Murray, D., McGrew, J. H., Brevoort, K., & Wong, V. W. (2018). A preliminary study of activation, stress, and self-management of parents of children with autism spectrum disorder. *Journal of Child and Family Studies,**27*, 825–834. 10.1007/s10826-017-0814-5

[CR37] Stadnick, N. A., Aarons, G. A., Martinez, K., Sklar, M., Coleman, K. J., Gizzo, D. P., Lane, E., Kuelbs, C. L., & Brookman-Frazee, L. (2022). Implementation outcomes from a pilot of “Access to Tailored Autism Integrated Care” for children with autism and mental health needs. *Autism,**26*(7), 1821–1832. 10.1177/13623613211065801 35083919 PMC9325918

[CR38] Vohra, R., Madhavan, S., Sambamoorthi, U., & St Peter, C. (2014). Access to services, quality of care, and family impact for children with autism, other developmental disabilities, and other mental health conditions. *Autism,**18*(7), 815–826. 10.1177/1362361313512902 24353274 PMC4908578

[CR39] Wood, J. J., McLeod, B. D., Klebanoff, S., & Brookman-Frazee, L. (2015). Toward the implementation of evidence-based interventions for youth with autism spectrum disorders in schools and community agencies. *Behavior Therapy,**46*(1), 83–95. 10.1016/j.beth.2014.07.003 25526837

[CR40] Yu, Y., Ruble, L., McGrew, J., & Murray, D. (2023). Parent Activation Measure for Developmental Disabilities (PAM-DD) in caregivers of individuals with ASD. *Journal of Autism and Developmental Disorders,**53*(1), 110–120. 10.1007/s10803-021-05263-7. Epub 2022 Jan 20 PMID: 35050439 PMCID: PMC9889422. 35050439 PMC9889422

